# Childhood Adversity and the Creative Experience in Adult Professional Performing Artists

**DOI:** 10.3389/fpsyg.2018.00111

**Published:** 2018-02-09

**Authors:** Paula Thomson, S. V. Jaque

**Affiliations:** ^1^Department of Kinesiology, California State University, Northridge, Los Angeles, CA, United States; ^2^York University, Toronto, ON, Canada

**Keywords:** adverse childhood experiences (ACEs), anxiety, creativity, flow, performing artists, shame, trauma

## Abstract

Childhood adversity is identified as any exposure to abuse, neglect or family dysfunction. Greater exposure to childhood adversity has been strongly identified with increased morbidity and mortality. The aim of this study was to examine differences in creative experiences, fantasy proneness, dispositional flow, exposure to adult traumatic events, and psychopathology (internalized shame, trait anxiety), amongst professional performing artists who experienced no childhood adversity, some adversity, or substantial adversity. This cross-section IRB approved study examined 234 professional performers (dancers, opera singers, actors, directors, musicians). Self-report measurements were included to examine the following psychological factors: adverse childhood experiences (ACEs), experience of creativity questionnaire, dispositional flow, trait anxiety, internalized shame, fantasy, and total adult and childhood traumatic events. The sample was divided into three groups based on ACE scores: 0 ACE (*n* = 93), 1–3 ACEs (*n* = 95), ≥4 ACEs (*n* = 42). The MANCOVA (with age and gender as covariates) results revealed no significant (*p* = 0.280) differences between all three ACE groups for the nine flow scales (optimal performance measurements). Performing artists with ≥4 ACEs had significantly stronger creative experiences (*p* = 0.006) related to distinct creative processing, absorption, and a transformational sense of self and the world. They were also more fantasy prone, shame-based, anxious, and experienced more cumulative past traumatic events (*p* < 0.001). Although the high ACE group experienced greater negative effects, they also endorsed positive creative performance experiences.

## Introduction

“This will be our reply to violence: to make music more intensely, more beautifully, more devotedly than ever before.”

(Bernstein, 1963)

Childhood adversity is identified as any exposure to abuse (emotional, physical, sexual), neglect (emotional, physical), and/or family dysfunction (parental separation/divorce, family member with mental illness and/or substance abuse, domestic violence, family member imprisoned) (Felitti and Anda, 2010). Typically, many of the adversity items co-occur, which increases the likelihood of later psychopathology (Kessler et al., 2010). As well, greater exposure to childhood adversity has been strongly identified with increased morbidity and mortality (Felitti et al., 1998; Felitti and Anda, 2010). According to the adverse childhood experiences (ACEs) study conducted by Felitti et al. (1998) at Kaiser Permanente’s Department of Preventive Medicine (San Diego) and Felitti and Anda (2010) at the Centers for Disease Control and Prevention (CDC), greater exposure to early childhood adversity is associated with multiple medical and psychiatric problems (Felitti and Anda, 2010). In international epidemiological studies, at least 40 million children are abused each year (Crimes Against Children Research Center, 2017). These high rates gathered by the ACE study and the epidemiological studies provide information about the general population; however, limited studies have focused on ACEs in performing artist samples. Along with childhood adversity, past adult trauma has been associated with intrusive emotional mental imagery (Holmes, 2003/2004) that may compromise general performance levels and increase anxiety. Such experiences, coupled with daily psychological and social uncertainty that typifies a performance career, are related to difficulty managing components of the creative process such as effectively meeting challenging tasks and solving problems, (Mittal and Griskevicius, 2014).

Engaging in the creative behavior of performance is often associated with an increased capacity for resilience and adaptability (Bennett, 2009; Thomson and Jaque, 2017). When engaging in a creative experience a distinct sense of pleasure, power, meaning, and purpose occur; however, creative experiences may also increase feelings of vulnerability and anxiety (Nelson and Rawlings, 2009). Based on phenomenological interview studies, the lived creative experiences of individuals who worked in diverse creative domains were identified. These experiences included distinct creative experiences, anxiety prior to the creative process, absorption during the creative experience, a sense of power and pleasure while creating, and clarity and preparation before and during the creative process. The phenomenological analyses also revealed three existential creative experiences; they are a sense of transformation of self and world, centrality of purpose and meaning, and a sense of working beyond the personal (Nelson and Rawlings, 2009).

Unlike creative experiences that evoke positive and negative emotions, optimal performances, or flow states, provide fully positive integrative experiences that are embedded in the activity (Kirchner et al., 2008). Optimal performance usually includes heightened awareness of skills, expressive goal setting and achieving these goals, and a capacity to focus and meet the performance challenge. Examining flow involves nine dimensions that contribute toward flow experiences. These dimensions include: challenge–skill balance, merging action and awareness, clear goal setting, unambiguous feedback while performing, undivided concentration on the task at hand, a sense of control while performing, a loss of self-consciousness, a sense of time transformation, and an autotelic experience (Csikszentmihalyi, 1990). For most performing artists, various dimensions of flow are frequently heightened. While most individuals experience some degree and frequency of flow, a smaller proportion possess a dispositional trait (autotelic personality) that enables them to repeatedly enjoy flow states (Csikszentmihalyi, 1990). Compared to a general population sample, higher flow states, as well as markers for an autotelic personality, have been found in performing artists (Thomson and Jaque, 2016). The capacity to achieve flow states and engage in creative experiences adds to the value and meaning derived from a career in the performing arts.

Engaging in fantasy is an integral component in the performing arts. Fantasy is considered to be an attentional shift from external to internal stimulation (McDaniel et al., 2000/2001). It is generally considered to be an adaptive and healthy aspect of psychological functioning. Although most performers engage in moderate levels of fantasy, some performers are regarded as high fantasy prone individuals. They are able to vividly imagine past memories and create new scenarios with full sensory and emotional details. They demonstrate richer and more complex storytelling (Merckelbach, 2004). These high fantasizers are generally well adjusted; however, some high fantasy prone individuals suffer greater psychopathology and experience more negative affect (Waldo and Merritt, 2000; Rauschenberger and Lynn, 2002/2003; Cuper and Lynch, 2008/2009).

Although positive experiences are generally associated with the performing arts, study findings suggest that performing artists also experience more vulnerability to psychopathology. For example, in a study that examined musicians they reported high rates of anxiety, depression, shame, substance abuse, and suicidality (Kenny and Asher, 2016). Studies that investigated dancers and actors found a similar array of psychopathology (Thomson and Jaque 2011, 2011/2012, 2012, 2015). This range of psychopathology is characteristic of acute and chronic stress, conditions that typify performing arts careers (Weber and Jaekel-Reinhart, 2000). As well, increased anxiety was associated with highly creative individuals (Carlsson, 2002). Many performing artists identify anxiety as a significant problem; they attributed decreased self-confidence, disruptions in performance skills, loss of pleasure and enjoyment, and chronic somatic symptoms such as those found in sleep or cardiovascular disorders to elevated anxiety (Sataloff et al., 1999; Langendorfer et al., 2006). Unfortunately, when these anxiety symptoms persist without relief, many performers opt to leave the profession (Yoshie et al., 2008).

Although performance anxiety is a common experience, exposure to public humiliation and rejection is equally common in performers. These experiences intensify an internalization of shame, a condition that manifests as negative beliefs about the worthiness of self in relationship to others (Nathanson, 1992). Shame is a self-conscious emotion (Nathanson, 1992; Schoenleber and Berenbaum, 2012). Unlike other negative emotions, shame involves the subjective experience that the self is defective. Shame is a highly painful mechanism that pulls the individual away from whatever might be of interest. It mobilizes behaviors of hiding and withdrawing and casts the individual into a primary state of isolation. Shame limits a capacity for intimacy and empathy (Nathanson, 1992). At toxic levels, shame can psychologically cripple an individual, evoking a sense of emptiness and an inner torment of the soul. In performing artists, elevated shame has been associated with diminished flow states and increased perfectionism and anxiety (Eusanio et al., 2014; Thomson and Jaque, 2016).

## Study Goals and Hypothesis

In this cross-sectional investigation, the aim of the study was to examine differences in adult professional performing artists who experienced no childhood adversity, one to three different childhood adversities, and four or more adversity types. Previous research findings indicate that exposure to four or more childhood adversity types exponentially increase the risk for later physical and psychological pathology (Felitti et al., 1998). In this study, negative psychological factors included trait anxiety, internalized shame and increased exposure to adult traumatic events. Fantasy proneness has been identified as a protective and risk factor for psychopathology so it was included with the psychological factors (Klinger et al., 2009). Positive psychological factors commonly associated with the performing arts included opportunities to experience creativity and flow states (Csikszentmihalyi, 1990; Nelson and Rawlings, 2009). These positive factors, along with fantasy proneness, were examined to balance the negative factors typically ascribed to individuals exposed to more childhood adversity. We hypothesized that more ACEs would be related to increased psychological difficulties (anxiety, shame, exposure to adult traumatic events) and decreased positive creative and performance flow experiences.

## Materials and Methods

### Participants and Procedures

This study was carried out in accordance with the recommendations of the American Psychological Association Ethics Guidelines and the Committee for the Protection of Human Subjects, Office of Research and Sponsored Projects. Written informed consent from all subjects was obtained. All subjects gave written consent in accordance with the Declaration of Helsinki. The protocol was approved by the Committee for the Protection of Human Subjects at California State University, Northridge under the leadership of Dr. Daniel Rastein. This cross-sectional study incorporated a sub-sample of 234 participants: 83 (36%) actors, directors, designers; 129 (55%) dancers; and 20 (9%) musicians and opera singers that were part of a larger psychophysiological study that investigated psychological and physiological effects of performance stress. The participants were drawn from University and Community groups. The mean age was 23.34, ranging from 59 to 18 years. There were 71 (30%) men and 163 (70%) women participating in the study. The major ethnicity groups included 34 (15%) African Americans, 47 (20%) Asians, 96 (41%) Caucasian, and 54 (23%) Latino. Criteria for professional status included: (1) five or more years of training, (2) performance experience in professional concert venues. General criteria for participation in the study included: (1) no orthopedic injury or illness, and (2) no physiological or psychological illness that would inhibit current participation in performance. There were no restrictions for gender, race or ethnicity.

All participants completed an informed consent form, a brief biographical screener, and seven self-report instruments to assess (1) adverse childhood experiences (2) creative experiences, (3) dispositional flow, (4) trait anxiety, (5) internalized shame, (6) fantasy proneness, and (7) adult traumatic events. These forms were completed in the studio or laboratory 2 weeks prior to a public performance. This timeline decision was based on a desire to measure negative psychological variables without the imminent stress of pending performances. For participants who participated in the test–re-test protocol, the self-report instruments were completed 6 months later in the laboratory.

### Measurements

#### Adverse Childhood Experiences (ACEs)

The ACE (Felitti and Anda, 2010) is a dichotomous 10 item self-report instrument that assesses categories of childhood abuse, neglect, and household dysfunctions. A total score of yes responses are derived, regardless of frequency or intensity. The abuse category probes for emotional, physical, and sexual abuse, the neglect category assesses emotional and physical neglect, and the household dysfunction category includes mother treated violently, substance abuse, parental separation or divorce, household member imprisoned, and/or suffering a mental illness. An example of an ACE question is “Did a parent or other adult in the household often…Swear at you, insult you, put you down, or humiliate you? or act in a way that made you afraid that you might be physically hurt? Yes/No.” To determine reliability, the ACE was administered a second time 6 months later to a smaller sample of participants. The result of the test–re-test reliability calculation was stable (*r* = 0.78, *p* < 0.01).

#### Dispositional Flow Scale-2 (DFS2)

The DFS2 (Jackson and Eklund, 2004) is a self-report, 36 item, instrument that assesses the construct of dispositional flow (performing in general). The DFS2 provides a single global flow score that is comprised of all 36 items. The nine flow dimensions, each with four items, contribute unequally to the global flow score (Jackson and Eklund, 2002; Jackson et al., 2008). A five-point Likert scale (*1 = never to 5 = always*) is used, with nine subscale scores measuring mean dimensional concepts of flow and a total mean scale score assessing a global flow construct. There is adequate reliability, construct validity and internal consistency in the DFS2. The Cronbach alpha scores in this study include global dispositional flow (α = 0.923), and the nine dimensional flow scales: (1) Challenge–Skill Balance (CSB: α = 0.826), (2) Merging of Action and Awareness (MAA: α = 0.808), (3) Clear Goals (CG: α = 0.317), (4) Unambiguous Feedback (UF: α = 0.879), (5) Concentration on the Task at Hand (CTAH: α = 0.898), (6) Sense of Control (SC: α = 0.918), (7) Loss of Self-Consciousness (LSC: α = 0.856), (8) Transformation of Time (TT: α = 0.858), and (9) Autotelic Experience (AE: α = 0.876). The flow scale scores can be divided: firstly, low agreement (scores ranging between 1 and 2) which suggests that the person’s experience was not substantially “flow-like” in nature, secondly, moderate level (scores ranging between 2 and 4) indicating some endorsement of flow experiences, and lastly, high level (scores ranging between 4 and 5) indicating the respondent endorsed frequent flow or always experienced flow in their selected activity. The scales have been used on individuals ranging from 16 to 82 years of age, with a minimum age of 15 recommended and no upper age range limits.

#### Experience of Creativity Questionnaire (ECQ)

This instrument was developed from qualitative research and was intended to offer a means to implement quantitative methods to gather information that is more phenomenologically rooted (Nelson and Rawlings, 2009). There are 44 questions probing for creative experiences and 19 questions examining existential experiences. The five-point Likert scale ranges from 1 (*definitely not my experience*) to 5 (*very much my experience*). Two sample questions from the ECQ include: “I put myself in the mood I wanted my creative work to take on.” and “I have found there is a compulsive, addictive quality to the experience of being engaged in the creative process.” It has adequate reliability as evidenced for each Cronbach alpha subscale score. Part A consists of 44 items assessing experiential dimensions: (1) Distinct Experience (α = 0.791); (2) Anxiety (α = 0.738); (3) Absorption (α = 0.836); (4) Power/Pleasure (α = 0.750); and (5) Clarity/Preparation (α = 0.637). Part B, a three scale and 19 item section, attempts to understand the existential dimensions of the creative process: (1) Transformation (α = 0.721); (2) Centrality (α = 0.786); and (3) Beyond the Personal (α = 0.697).

#### Internalized Shame Scale (ISS)

The ISS (Cook, 2001) is a 30 item five-point Likert-type frequency self-report scale, ranging from 0 (*never*) to 4 (*almost always*). There are 24 items that measure internalized shame and six items that measure self-esteem. The shame scale focuses on both negative emotions and cognitive states and incorporates high intensity wording that is associated with internalized shame. On the shame scale, scores ≤49 suggest low shame, scores between 50 and 59 indicate frequent shame experiences, and scores ≥60 are considered high (toxic) shame. Gender and age were found not to be factors in interpreting ISS scores. The ISS is a highly valid instrument that can discriminate clinical from non-clinical samples. It demonstrates a pattern of elevated shame in clinical samples that suffer from childhood sexual abuse, substance abuse disorders, eating disorders, family of origin dysfunction, and insecure attachment classifications. In the convergent and divergent validity studies, the ISS had strong associations with other measures of self-concept, social desirability, and psychopathology (including depression, anxiety, suicidality probability, anxiety, and anger) (Vikan et al., 2010). In this study, the ISS had excellent internal consistency (α = 0.966).

#### Inventory of Childhood Memories and Imaginings (ICMI)

The ICMI is a self-report instrument that was developed by Wilson and Barber (Wilson and Barber, 1983; Lynn and Rhue, 1988). It is a dichotomous, paper-and-pencil questionnaire, consisting of 52 items that probe for experiences and memories from childhood and during the present time, such as; “when I was a child I enjoyed fairytales, at the present time I am very imaginative,” “when I was a child I lived in a make-believe world, as an adult I still occasionally live in a make-believe world.” The scoring is a simple total of all items endorsed by the subject. This instrument has adequate reliability and validity and discriminates between high fantasy-prone (scores above 40), medium fantasy-prone (scores between 11 and 39) and low fantasy-prone (scores below 10). In this study, the Cronbach’s alpha was high α = 0.874.

#### Trait Anxiety (STAI-Y2)

The STAI-Y2 is a 20 item Likert intensity measure (Spielberger, 1983). Trait anxiety items are rated on a four-point scale (e.g., from *Almost Never* to *Almost Always*). Trait anxiety can be defined as feelings of stress, worry, or discomfort that one experiences on a day-to-day basis. Higher scores indicate greater anxiety. Internal consistency and test–re-test reliability are excellent (Spielberger, 1983). In this study, Cronbach’s alpha was high α = 0.932.

#### Traumatic Events Questionnaire (TEQ)

A self-report 11 item dichotomously scored instrument (Vrana and Lauterbach, 1994; Lauterbach and Vrana, 2001) assessed exposure to nine different traumatic events (accidents, natural disasters, crime, child abuse, rape, adult abusive experiences, witnessing death/mutilation of someone, being in a dangerous/life-threatening situation, and receiving news of an unexpected death of a loved one). The final two items probed for any other traumatic event not listed, and for traumatic event(s) that were too difficult to discuss with anyone. Since calculating the internal consistency of the TEQ was inappropriate, a test–re-test reliability was calculated 6 months later in a subsample of participants; between the two TEQ scores there was high correlation (*r* = 0.874, *p* < 0.001).

### Analyses

SPSS 24 was used for all calculations. First, descriptive statistical analyses were conducted. Participants were then assigned to one of three groups based on their ACE score: No ACE (*n* = 94); 1–3 ACEs (*n* = 96); and ≥4 ACEs (*n* = 42). Chi Square analyses were conducted to examine group distribution differences between the original Kaiser Permanente study of a general population and the performing artist group who participated in this study. To further analyze group differences, multivariate analyses of covariance (MANCOVA) were conducted (with age and gender as the covariates). The three MANCOVA analyses were determined based on discrete variable topics. Specifically, they were organized based on discrete creative experiences, flow dimensions, and potential psychopathology factors. The first MANCOVA was calculated to examine whether there were significant between group effects for flow (global and nine dimension scales). A second MANCOVA was calculated to address group differences for experiences of creativity. In the third MANCOVA, group differences were examined for fantasy proneness, trait anxiety, internalized shame, and traumatic events. Gender and age were included as covariates in all MANCOVA calculations to account for any effects of gender and age on these variables. We also wanted to account for the fact that there were more women in the sample. In the MANCOVA analyses, Bonferroni alpha (0.05) corrections were used to determine the nature of the differences between the group means.

## Results

The descriptive and Chi Square statistics demonstrated that performing artists experienced significantly higher rates of emotional abuse and neglect compared to the original ACE study (Felitti et al., 1998). Although not statistically significant they also experienced higher rates of exposure to parental separation/divorce and family member imprisonment. They experienced lower prevalence rates for all other ACE items. See **Table 1** for details.

**Table 1:** Number and percentage of participants (and chi square statistics) who experienced ACE items and total ACEs compared to the original ACE Kaiser Permanente study (Felitti et al., 1998).

Category	Full sample	ACE study	χ^2^
ACE #1 Abuse–Emotional	60 (24.9%)	10.6%	6.64**
ACE #2 Abuse–Physical	34 (14.7%)	28.3%	5.01*
ACE #3 Abuse–Sexual	26 (11.2%)	20.7%	3.72*
ACE #4 Neglect–Emotional	43 (18.5%)	14.8%	0.57
ACE #5 Neglect–Physical	8 (3.4%)	9.9%	4.03*
ACE #6 Family dysfunction–Separated/divorced	84 (36.2%)	23.3%	4.06*
ACE #7 Family dysfunction–Domestic violence	24 (10.3%)	12.7%	0.44
ACE #8 Family dysfunction–Addiction	38 (16.4%)	26.9%	3.58
ACE #9 Family dysfunction–Mental illness	38 (16.4%)	19.4%	0.32
ACE #10 Family dysfunction–Prison	16 (6.9%)	4.7%	0.35
No ACEs	94 (40.5%)	31.3%	2.17
1–3 ACE	96 (41.4%)	57.8%	5.78*
≥4 ACEs	42 (18.1%)	26.5%	2.32

*χ^2^, Chi Square. ^∗^*p* < 0.05; ^∗∗^*p* < 0.01*.

In the first MANCOVA (age and gender covariates) calculation, group differences were examined for flow states; Bonferroni alpha (0.05) corrections were applied and reported. In this analysis, no significant differences were found: Wilks’s Λ = 0.908, *F*(18,430) = 1.173, *p* = 0.280, η^2^ = 0.047; gender, *p* < 0.001; age, *p* = 0.031. See **Table 2** for details.

**Table 2:** Group mean descriptive statistics, standard deviations (SD), and MANCOVA analysis for flow dimension in the three ACE groupings.

	0 ACE	1–3 ACE	≥4 ACE
Global flow	3.87 (0.60)	3.85 (0.59)	3.80 (0.55)
CSB	3.93 (0.71)	3.92 (0.68)	3.92 (0.67)
MAA	3.62 (0.75)	3.65 (0.77)	3.65 (0.68)
CG	4.23 (1.53)	4.12 (0.66)	4.17 (0.69)
UF	4.03 (0.67)	3.95 (0.70)	3.93 (0.67)
CTAH	3.95 (0.70)	3.87 (0.76)	3.79 (0.88)
SC	3.96 (0.68)	3.92 (0.79)	3.73 (0.76)
LSC	3.33 (0.97)	3.12 (1.01)	3.05 (1.13)
TT	3.77 (0.91)	3.63 (0.89)	3.57 (0.79)
AE	4.35 (0.63)	4.43 (0.70)	4.43 (0.61)

*CSB, challenge–skill balance; MAA, merging action and awareness; CG, clear goals; UF, unambiguous feedback; CTAH, challenge for the task at hand; SC, self-control; LSC, loss of self-consciousness; TT, transformation of time; AE, autotelic experiences. MANCOVA (age and gender covariates) comparison of mean scores showing significant group differences: Wilks’s Λ = 0.908, *F*(18,430) = 1.173, *p* = 0.280, η^*2*^ = 0.047; gender, *p* < 0.001; age, *p* = 0.031.*

In the second MANCOVA (age and gender covariates) calculation, group differences were examined for creative experiences; Bonferroni alpha (0.05) corrections were applied and reported. Significant differences were found: Wilks’s Λ = 0.837, *F*(16,372) = 2.157, *p* = 0.006, η^2^ = 0.085; gender, *p* = 0.250; age, *p* = 0.096. In the pairwise comparisons, significant differences were found for the following scales: (1) Distinct Experiences (no ACE and ≥4 ACEs: *p* = 0.031; 1–3 ACEs and ≥4 ACEs: *p* = 0.044); (2) Absorption (no ACE and ≥4 ACEs: *p* = 0.008; 1–3 ACEs and ≥4 ACEs: *p* = 0.025); and (3) Transformation (no ACE and ≥4 ACEs: *p* = 0.01). See **Table 3** for details. This analysis demonstrated that performing artists who were exposed to four or more childhood adversity types experienced more intense creative and existential experiences compared to the groups with no childhood experience or one to three exposure types. Significant differences were found for the following factors: distinct experience, absorption, and transformation, with the high exposed performers endorsing more creative intensity compared to performers with minimal or no childhood adversity.

**Table 3:** Group mean descriptive statistics, standard deviations (SD), and MANCOVA analysis for creative and existential experiences in the three ACE groupings.

	0 ACE	1–3 ACE	≥4 ACE
Power–Pleasure	39.04 (8.27)	38.28 (8.98)	41.68 (9.46)
Distinct experience	27.63 (7.60)	28.19 (7.71)	31.68 (6.24)^∗^
Absorption	32.87 (8.66)	33.85 (8.89)	38.30 (7.44)^∗∗^
Anxiety	18.46 (6.09)	18.65 (6.06)	20.41 (4.49)
Clarity	14.17 (3.60)	14.54 (4.83)	15.24 (3.48)
Certainty	30.39 (9.87)	30.30 (9.27)	33.78 (7.91)
Transformation	24.11 (7.39)	25.30 (7.21)	28.43 (6.39)^∗^
Beyond personal	9.33 (3.45)	10.00 (3.37)	10.14 (3.30)

*MANCOVA (age and gender covariates) comparison of mean scores showing significant group differences: Wilks’s Λ = 0.837, *F*(16,372) = 2.157, *p* = 0.006, η^*2*^ = 0.085; gender, *p* = 0.250; age, *p* = 0.096. See results for narrative details on pairwise comparison differences. ^∗^*p* < 0.05; ^∗∗^*p* < 0.01*.

In the third MANCOVA (age and gender covariates) calculation, group differences were examined for psychopathology variables; Bonferroni alpha (0.05) corrections were applied and reported. Significant differences were found: Wilks’s Λ = 0.760, *F*(8,444) = 8.173, *p* < 0.001, η^2^ = 0.128; gender, *p* = 0.674; age, *p* = 0.141. Significant results were found in the pairwise comparison analyses: (1) Anxiety (no ACE and 1–3 ACEs: *p* = 0.017; No ACE and ≥4 ACEs: *p* < 0.001); (2) Shame (no ACEs and 1–3 ACEs: *p* = 0.001; No ACE and ≥4 ACEs: *p* < 0.001); (3) Fantasy Proneness (no ACE and 1–3 ACEs: *p* = 0.014; No ACE and ≥4 ACEs: *p* < 0.001); and (4) traumatic events (TEQ) (no ACE and ≥4 ACEs: *p* < 0.001; 1–3 ACEs and ≥4 ACEs: *p* < 0.001). See **Table 4** for details. In this MANCOVA analysis, a significant dose effect was demonstrated for psychological factors. Performers with no childhood adversity had significantly lower scores compared to performers with one to three exposure types and to performers with four or more exposure types. When examining total adult traumatic events, there was a significant increase in rates between each group, a finding that reinforces previous research demonstrating higher rates of re-traumatization in adulthood for individuals exposed to more cumulative childhood adversity (Felitti and Anda, 2010).

**Table 4:** Group mean descriptive statistics, standard deviations (SD), and MANCOVA analysis for psychological factors in the three ACE groupings.

	0 ACE	1–3 ACE	≥4 ACE
Anxiety	35.42 (10.29)	40.02 (11.42)	43.79 (11.52)^∗^
Shame	19.08 (17.45)	26.73 (19.68)	32.24 (20.61)^∗^
Fantasy	19.25 (9.02)	22.84 (7.80)	26.88 (8.66)^∗^
TEQ	1.24 (1.48)	1.80 (1.77)	3.60 (2.60)^∗^

*TEQ, Traumatic Events Questionnaire. MANCOVA (age and gender covariates) comparison of mean scores showing significant group differences: Wilks’s Λ = 0.760, *F*(8,444) = 8.173, *p* < 0.001, η^*2*^ = 0.128; gender, *p* = 0.674; age, *p* = 0.141. See results section for narrative details on pairwise comparison differences. ^∗^*p* < 0.001*.

## Discussion

The study findings demonstrated that despite exposure to four or more childhood adversity types, these performing artists had significantly greater distinct creative experiences; in particular, they endorsed more intense creative processing in comparison to those with no childhood adversity or some adversity (less than four items on the ACE). Based on higher scores on the distinct creative experience scale, the performers in the high ACE group were more aware of a loss of a sense of self, a greater sense of contact with a force beyond themselves, greater emotional intensity that coexisted with emotional stability, a heightened awareness of technical and expressive abilities, and increased spiritual awareness during the creative process (Nelson and Rawlings, 2009). This finding suggests that performing artists with four or more ACEs are more aware of the creative process; it may indicate that despite more exposure to childhood adversity, they are able to recognize and value the creative process (Drus et al., 2014). This group also endorsed deeper states of absorption, including heightened awareness of a state of inspiration and a sense of discovery during the creative process. They were better able to be receptive to the artwork, plus they were able to freely move between absorption states and the distancing process required during critical awareness (Nelson and Rawlings, 2009). Lastly, the high ACE group identified greater appreciation for the transformational quality of creativity, in particular, how the creative process enabled a deeper engagement with the self and world. They recognized that it operated as a powerful force in their life.

There were no differences between the three ACE groups for the other dimensions of the creative process. These included anxiety, which examines a sense of vulnerability during the creative process as well as anxiety that is activated before and at the start of the process. All three groups equally experienced power and pleasure; specifically they experienced a sense of control, power, and pleasure during the analytic aspect of the creative process. Clarity and preparation is a subscale that assesses feelings of certainty and clarity about the direction of the creative work and how the work elicits moods related to the work; all three groups enjoyed a sense of clarity and preparation. In the existential subscales, there were no group differences on the subscale, centrality, a scale that measures meaning garnered while engaging in the creative process. A feeling of creative centrality is associated with a spiritual and healing component in the performer’s life; it gives a sense of purpose. There were no group differences for a sense of operating beyond the personal; this creative component is a process of meaning making that connects the creative work and the creator with a larger intersubjective relational field and gives an interpersonal context of the performance (Nelson and Rawlings, 2009).

Regardless of ACE exposure, performing artists in this sample were able to experience all flow dimensions with equal frequency and intensity. The lack of difference between the three groups may be related to performance training that enhances flow skills (Jackson et al., 2001). The participants in all three ACE groups were able to find a balance between the perceived challenge of the performance activity and their skill level required to optimally meet the challenge. The performers in all three ACE groups were able to merge action and awareness, which is the ability to become absorbed while maintaining awareness of skill execution. All participants, regardless of ACE exposure, were able to establish appropriate goal setting, especially when they were able to define a goal and sustain the preparation required to achieve that goal. ACE exposure did not compromise an ability to unambiguously interpret feedback that helps clarify goal achievement. They were equally able to receive internal and/or external feedback necessary to modify and optimize performance. All three groups endorsed similar abilities to fully concentrate on the task at hand; they were able to block extraneous thoughts and distractions while maintaining awareness of the present performance moment. Likewise, the participants in all three ACE groups maintained a sense of self-control while losing self-consciousness; they were equally able to quiet internal doubts and criticism. All three ACE groups shared similar perceptions of transformation of time, especially when a sense of self was no longer subjected to ongoing self-evaluation. Lastly, an autotelic experience, or an intrinsically rewarding experience related to an autotelic personality, was equally recognized in performers who had no ACE exposure, some exposure, and high exposure.

In general, the three ACE groups did not differ for flow experiences during performance. Surprisingly, the creative process was intensified and valued by performers in the high ACE exposure group. Unfortunately, this high ACE group also had higher negative psychological factors, specifically greater trait anxiety, internalized shame, and more cumulative past traumatic events. They were also more fantasy prone, a factor that may enhance creativity (Lack et al., 2003), as well as intensify the subjective experience of anxiety and shame (Thomson et al., 2009; Thomson and Jaque, 2011/2012). Struggling with increased shame and anxiety, coupled with managing more exposure to adult traumatic experiences, adds to the stress of a performing arts career (Thomson and Jaque, 2017); however, these factors can also enhance creativity (Akinola and Mendes, 2008). Creativity is also associated with enhanced executive functioning, resilience, and optimal functioning (Mittal et al., 2015). It is heartening to know that despite negative factors the performers with high ACE exposure in this study enjoyed the creative performance experience, which may indicate more creative resilience.

Lastly, when comparing the performing artist group to the original general Kaiser Permanente sample, performing artists were exposed to significantly more emotional abuse, as well as more parental separation/divorce. Emotional abuse is marked by inconsistent or inappropriate interactions with a child, as well as failure to recognize the individuality of the child and to promote the child’s socializing needs (Glaser, 2002). This form of adversity insidiously diminishes self-concept and increases shame, anxiety, anger, depression, self-harming behaviors, eating disorders, and personality disorders (Taillieu et al., 2016).

The limitations in this study include the inherent bias contained in self-report measurements. Generalizability to other populations is limited because all participants were professional performing artists. The uneven gender distribution also limits generalizability; although gender was included as a covariate in all the statistical analyses. It is recommended that future studies examine gender differences for ACE distribution as well as the positive and negative psychological factors that were examined in this study. Determining factors that may buffer the effects of ACEs is important. Conducting structural equation modeling to evaluate mediating and moderating effects would add to future studies.

## Conclusion

Although the high ACE group experienced greater negative psychological effects, they also endorsed positive creative performance experiences. Providing optimal environments to train young performers is critical; however, frequently these training environments can be traumatizing. For example, imposing expectations on children that are developmentally inappropriate strains their developing stress systems (Romens et al., 2015). If teachers or coaches are verbally or physically aggressive then the training environments they create are not much different than adverse family environments. Traumatized talented children may manifest atypical stress reactions. Training children in environments that focus on intense evaluation and criticism may compromise their ability to perform creatively (Byron et al., 2010). During training, they may respond with a decreased sense of control, including withdrawing from challenging tasks. They either withdraw from performance training or become hyper-vigilant to potential training threats. This will compromise the realization of their talent (Mittal and Griskevicius, 2014).

Performers in this study identified frequent flow experiences, a finding that indicated they valued the positive integrative experience of performing (Csikszentmihalyi, 1990). Performers with four or more childhood adversity types also endorsed more intense creative experiences, despite the fact that they experienced more trait anxiety, internalized shame, fantasy proneness, and higher rates of adult trauma. This study demonstrated that 18% of performers had elevated childhood trauma exposure. It is essential to support all performers, including those with substantial trauma history, so that they thrive in their professional careers (Nagel, 2009).

## Author Contributions

All authors listed have made a substantial, direct and intellectual contribution to the work, and approved it for publication.

## Conflict of Interest Statement

The authors declare that the research was conducted in the absence of any commercial or financial relationships that could be construed as a potential conflict of interest.
